# Characteristics of the First 284 Patients Infected with the SARS-CoV-2 Omicron BA.2 Subvariant at a Single Center in the Apulia Region of Italy, January–March 2022

**DOI:** 10.3390/vaccines10050674

**Published:** 2022-04-24

**Authors:** Daniela Loconsole, Francesca Centrone, Anna Sallustio, Marisa Accogli, Daniele Casulli, Davide Sacco, Riccardo Zagaria, Caterina Morcavallo, Maria Chironna

**Affiliations:** 1 Hygiene Section, Department of Interdisciplinary Medicine, University of Bari, 70124 Bari, Italy; daniela.loconsole@uniba.it (D.L.); davide.sacco@uniba.it (D.S.); riccardo.zagaria1@uniba.it (R.Z.); 2 Hygiene Section, Department of Biomedical Sciences and Human Oncology-Hygiene Section, University of Bari, 70124 Bari, Italy; francesca.centrone@uniba.it (F.C.); marisa.accogli@uniba.it (M.A.); caterina.morcavallo@uniba.it (C.M.); 3 Hygiene Unit, Azienda Ospedaliero-Universitaria Consorziale Policlinico di Bari, 70124 Bari, Italy; annasallustio@libero.it (A.S.); daniele.casulli@policlinico.ba.it (D.C.)

**Keywords:** SARS-CoV-2 Omicron BA.2 subvariant, SARS-CoV-2 Omicron BA.1 subvariant, COVID-19 vaccine, surveillance, whole-genome sequencing, Italy

## Abstract

Since its initial detection, the SARS-CoV-2 Omicron sublineage BA.2 has been spreading rapidly worldwide. The aims of this study were to describe the first 284 patients infected with the Omicron BA.2 variant of concern (VOC) in the Apulia region of southern Italy and to assess the differences in the demographic and clinical characteristics of patients infected with the SARS-CoV-2 BA.1 and BA.2 variants. The demographic characteristics of patients, as well as information about symptoms, vaccinations and hospitalizations for COVID-19, were collected. A subset of samples from patients infected with the BA.2 variant was subjected to whole-genome sequencing. The characteristics of the first 284 patients infected with Omicron BA.2 and the first 175 patients infected with Omicron BA.1 were compared. The proportion of patients infected with the BA.2 variant rapidly increased, from 0.5% during the third week of 2022 to 29.6% during the tenth week of 2022. Ten isolates (out of 34 BA.2 isolates) contain the substitutional mutation, H78K in ORF3a, and four isolates include two mutations, A2909V in ORF1a and L140F in ORDF3a. Compared with patients infected with BA.1, those infected with BA.2 were more likely to be symptomatic and booster-vaccinated, and showed a shorter time from the last dose of vaccine to infection. The high transmissibility and immune-evasive properties of Omicron BA.2, which will become the leading SARS-CoV-2 VOC, suggest that short-term public health measures should not be discontinued in Italy.

## 1. Introduction

The SARS-CoV-2 Omicron variant of concern (VOC), first detected in Africa in November 2021, has rapidly spread worldwide within a few weeks [1]. Due to its significant growth advantage, higher secondary attack rates, greater ability to reproduce and higher vaccine breakthrough rate, the Omicron VOC has rapidly replaced the Delta VOC, becoming the dominating variant globally [1,2,3]. The Omicron variant includes three sublineages, termed BA.1, BA.2 and BA.3. Although the BA.1 lineage has been the most dominant to date, recent trends in India, South Africa, the United Kingdom and Denmark suggest that the proportion of patients infected with BA.2 is increasing [1]. In Denmark, BA.2 has rapidly replaced the BA.1 Omicron VOC as the dominant sublineage [4,5]. As of 18 January 2022, 79.4% of the BA.2 sequences submitted to the GISAID database were from patients in Denmark [5].

BA.2 differs from BA.1 in its genetic sequence, sharing 32 mutations with BA.1 but showing 28 distinct mutations. In particular, the BA.2 lineage does not harbor the 69/70 deletion in the spike protein. This mutation impairs the detection of the S gene by common commercial real-time PCR kits, a phenomenon called S gene target failure (SGTF), and was used as a proxy for early identification of the Alpha and Omicron VOCs [6,7,8,9]. BA.2 is thought to originate from overlooked virus evolution in individuals with low access to viral diagnosis and sequencing, in immunocompromised, chronically-infected patients, or in animals [10].

BA.2 is about 1.5-fold more contagious than BA.1 and 4.2-fold more contagious than Delta [3,11]. Moreover, BA.2 has a 30% greater potential than BA.1 to escape current vaccines, despite results showing that the loss in neutralizing activity of current mRNA vaccines is comparable to that already reported for BA.1 [11,12]. Clinical manifestations of infection with the BA.1 and BA.2 Omicron VOCs are similar [4]. Although reinfection with BA.2 following infection with BA.1 has been documented, initial data from population-level reinfection studies suggest that infection with BA.1 provides strong protection against reinfection with BA.2 [13,14].

In Italy, the first cases of infection with the Omicron VOC were identified at the end of November 2021, with its prevalence rapidly increasing, reaching 99.1% at the end of January 2022 [15]. During a 2-week period in January 2022, the prevalence of BA.2 in sequenced samples of patients infected with the Omicron VOC increased from 1% to 3% [15]. In the Apulia region of Italy, the prevalence of the Omicron VOC was 100% within a few weeks of its first identification in December 2021, with this virus inducing autochthonous outbreaks in booster-vaccinated subjects [9]. The first Omicron BA.2 subvariant in Apulia was identified in a sample collected on 31 January 2022 [16]. 

The present study sought to gain insight into the spread and molecular characteristics of the first 284 patients infected with the BA.2 Omicron VOC in the Apulia region of southern Italy. Moreover, a comparison of demographic and clinical characteristics between patients infected with the SARS-CoV-2 BA.1 and BA.2 variants has been performed.

## 2. Materials and Methods

Since the beginning of the COVID-19 pandemic, an integrated epidemiological and virological surveillance has been set up in Italy. All epidemiological and molecular data of cases are collected using an online national surveillance platform [17]. 

The present study included all SARS-CoV-2-positive samples collected from newly diagnosed patients analyzed at the Laboratory of Molecular Epidemiology and Public Health of the Hygiene Unit (A.O.U.C. Policlinico Bari, Italy) between 17 January 2022 and 13 March 2022. In the considered period, the estimated prevalence of the Omicron variant was 95.8% in Italy and 100% in the Apulia region [15]. The Laboratory of Molecular Epidemiology and Public Health is the coordinator of the Regional Laboratory Network for SARS-CoV-2 diagnosis in the Apulia region and the regional reference center, covering 60% of the province of Bari (1,230,205 inhabitants). During the study period, this laboratory processed an average of 3000 nasopharyngeal swabs per day, collected from both hospitalized and non-hospitalized patients. Nasopharyngeal swabs (UTM, FLOQ Swabs TM, Copan Italia, Brescia, Italy) were subjected to a commercial real-time PCR assay, as previously described [8]. Because the Delta variant was regarded as being completely replaced by the Omicron VOC during the study period, all SGTF-negative samples were assumed to be positive for BA.2. To confirm that SGTF negativity was an accurate indicator of BA.2 infection, a subset of the SGTF-negative samples was subjected to whole-genome sequencing (WGS), as described [18]. Phylogenetic analysis to investigate the relationships among strains of the BA.2 lineage was performed using the Nextclade sequence analysis web app [19]. Several Italian BA.2 strains identified during the study period and deposited in the GISAID database were included in the analysis [16]. 

The demographic characteristics of patients, as well as information about their symptoms, vaccinations and hospitalizations for COVID-19, were collected from the national surveillance platform [17]. Clinical cases were classified according to the Italian COVID-19 surveillance system as asymptomatic, mild infection in presence of fever < 38 °C, cough, malaise, gastrointestinal symptoms without fever; moderate infection with fever ≥ 38 °C, upper respiratory symptoms, anosmia/ageusia, gastrointestinal symptoms; severe infection in presence of fever ≥ 38 °C with dyspnea and lower respiratory tract infection. [17]. The clinical condition of each patient at diagnosis was recorded. Patients were classified as unvaccinated if no vaccine doses were administrated or if ≤14 days have elapsed since the first dose of any COVID-19 vaccine; partially vaccinated if >14 days have elapsed after the first dose of any COVID-19 two-dose vaccine or ≤14 days after completing the vaccination schedule for a single-shot or two-dose vaccine; fully vaccinated with primary series if >14 days have elapsed after a single-shot vaccine or after the second shot of a two-dose vaccine; fully vaccinated with primary series plus booster dose if >14 days have elapsed after the administration of the booster dose [20]. In the Apulia region, the majority of the population was vaccinated with mRNA vaccines.

Information on the first 284 patients infected with Omicron BA.2 was compared with information on the first 175 patients infected with Omicron BA.1 in the Apulia region from 8 December 2021 to 23 December 2021. Since the Omicron VOC was first identified in the Apulia region, it has been mandatory to notify the Ministry of Health of all suspected/confirmed Omicron VOC cases. Omicron VOC infection was suspected if a SGTF-positive sample was identified and was confirmed by whole-genome sequencing [21]. Statistical analyses were performed with STATA 12.0 software. Proportions and their 95% confidence intervals (CIs) were reported and compared by Chi-squared or Fisher’s exact tests, and mean times from the last dose of vaccine to infection with BA.2 and BA.1 VOCs were compared by *t*-tests. A *p*-value < 0.05 was considered statistically significant. 

Ethics committee approval was not required because the activities described in this study were part of the legislated mandate of the Health Promotion and Public Health Department of the Apulia region (Italy). All research involving human subjects was performed in accordance with the Declaration of Helsinki, as revised in 2013.

## 3. Results

**Figure 1:**
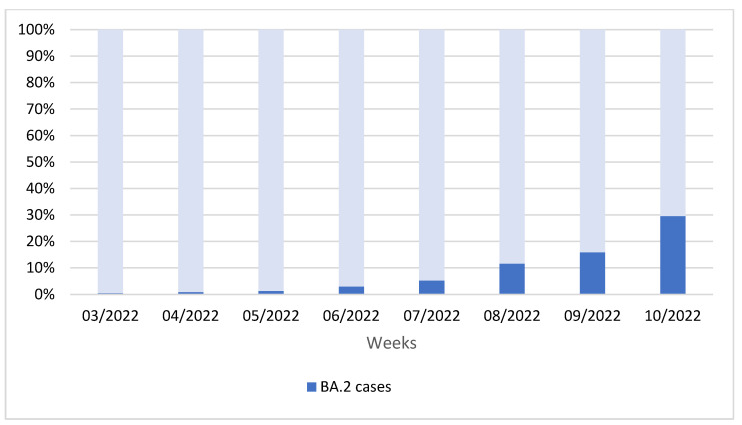
Distribution (%) of Omicron BA.2 cases among new SARS-CoV-2 infections identified from week 3 to week 10 of 2022 in the Apulia region of Italy.

Overall, 6109 new SARS-CoV-2 infections were diagnosed at the Laboratory of Molecular Epidemiology and Public Health of the Policlinico Hospital of Bari from 17 January to 13 March 2022. Among these newly diagnosed cases, 284 (4.6%) were negative for SGTF and were presumed to be caused by the BA.2 Omicron variant. The proportion of patients infected with BA.2 increased rapidly over time, from 0.5% during the third week of 2022 to 29.6% during the tenth week of 2022 (Figure 1).

The 284 patients infected with the Omicron BA.2 variant included 156 (54.9%) females and 128 (45.1%) males, of median age 42 years (interquartile range [IQR]: 27–60 years). Of these patients, 13 (4.6%), 19 (6.7%), 14 (4.9%) and 238 (83.8%) were aged 0–4, 5–11, 12–17 and ≥18 years, respectively. Of these 284 patients, 121 (42.6%) were symptomatic, nine (3.2%) had underlying chronic conditions, 66 (23.2%) required hospitalization and five (1.7%) died. Evaluation of vaccination status showed that 158 patients (55.6%) were fully vaccinated with primary series plus a booster dose, 54 (19.0%) were fully vaccinated with the primary series, 72 (25.4%) were unvaccinated and none (0%) were partially-vaccinated. Fifteen patients (5.3%), only two of whom were booster-vaccinated, experienced SARS-CoV-2 reinfection. The number of reinfections increased over time, from 0.06% during week 6 of 2022 to 2.2% during week 10. None of these patients experienced severe disease, although six required hospitalization. 

**Figure 2:**
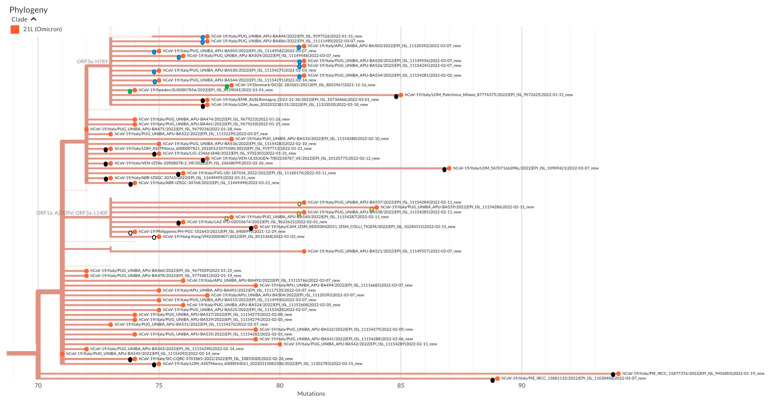
Phylogenetic tree of 60 SARS-CoV-2 Omicron BA.2 sublineage VOC based on their full genome sequences, including the 34 genomes examined in this study. Clade information using the GISAID and Nextstrain nomenclatures is shown. Blue dots indicate the Apulian sequences harboring the H78Y mutation in ORF3a; yellow dots indicate the Apulian sequences harboring the mutations A2909V in ORF1a and L140F in ORF3a; orange dots indicate other Apulian sequences; black dots indicate the Italian sequences included in the analysis; green dots indicate sequences from Denmark/Sweden; and white dots indicate sequences from the Philippines/Hong Kong.

Of the 284 samples, 34 (12.0%) were subjected to WGS. All 34 isolates were found to be BA.2-lineage Omicron VOC; their whole-genome sequences have been deposited in the GISAID database. Phylogenetic analysis of these 34 isolates showed that ten out of 34 BA.2 isolates contained the H78K substitution in ORF3a, a characteristic of the Sweden/Denmark BA.2 lineage, and four were positive for the mutations A2909V in ORF1a and L140F in ORF3a, which have been identified as characteristic of viruses of the Philippines/Hong Kong BA.2 lineage (Figure 2) [5]. There was no correlation between the presence of such mutations and the clinical status of the patients.

From 8 December to 23 December 2021, 175 persons suspected or confirmed as being infected with the BA.1 Omicron VOC were identified in the Apulia region and reported to the Ministry of Health, according to national guidelines [21]. Table 1 shows a comparison of the demographic and clinical characteristics of the patients infected with BA.2 and BA.1 Omicron VOCs.

The proportion of asymptomatic individuals was significantly higher in patients infected with BA.1 than BA.2 (*p* < 0.001). Moreover, patients infected with BA.2 were more likely to be fully vaccinated with the primary series plus a booster dose, whereas patients infected with BA.1 were more likely to be fully vaccinated with the primary series (*p* < 0.001). The average time from the last vaccine dose to infection was significantly longer in the BA.1 group (*p* = 0.01). Of the BA.2 hospitalized patients (*n* = 66), 12 (18.2%) showed severe symptoms and, of these, six were fully vaccinated with primary series plus a booster dose. Of the BA.1 hospitalized patients (*n* = 29), none showed severe symptoms. There were no significant differences in any other characteristics.

## 4. Discussion

The SARS-CoV-2 Omicron variant BA.2 lineage has been spreading rapidly worldwide since its first detection. As of February 2022, BA.2 genome sequences represented about 1% of Omicron genome submitted to the GISAID [5,16,22]. The rapid increase in patients infected with BA.2 in Denmark suggests that this epidemiological change may occur in other countries [3,5]. Also, the estimated prevalence of the Omicron BA.2 variant has increased rapidly in Italy and in the Apulia region within a short time, with BA.2 expected to completely replace the Omicron BA.1 VOC within a few weeks [23].

The median age of patients infected with BA.2 in the Apulia region was higher than that reported for patients infected with BA.2 in Denmark (32 years), a finding that may reflect differences in population distributions [4]. Moreover, older subjects vaccinated early in 2021 may have experienced a reduction in vaccine effectiveness due to waning immunity. The proportion of reinfected patients in the present study did not differ from that reported for Danish patients infected with BA.2 [4]. In a recent report, vaccinated persons presumably infected with BA.1 showed robust neutralizing antibody titers against BA.2, suggesting a cross-reactive natural immunity [24]. The increasing frequency of BA.2 infection in the context of the BA.1 surge is, therefore, likely associated with increased viral transmissibility rather than enhanced immunologic escape [24].

To date, five subgroups of BA.2 viruses have been identified, with these mutations thought to be specific to individual geographical areas [5]. Of the 34 samples sequenced in the Apulia region, ten harbored the Danish mutation and four harbored the Philippines/Hong Kong mutation. However, it was not possible to identify the origins of these infections or the presence of an epidemiological link, as the surge in cases impaired contact tracing.

The secondary attack rate of BA.2 Omicron VOC in the households has been estimated at 39% [3]. Moreover, unvaccinated individuals (OR: 2.19), fully vaccinated individuals (OR: 2.45) and booster-vaccinated individuals (OR: 2.99) were all more susceptible to BA.2 than to BA.1 infection [3]. These results may explain the new surge in SARS-CoV-2 infections in Italy and the Apulia region, suggesting that a fifth wave of COVID-19 is underway [23]. Despite BA.2 having greater transmissibility and immune-evasive properties, its transmissibility from vaccinated individuals with breakthrough infections is no higher than that of BA.1 [3]. The proportions of fully vaccinated and booster-vaccinated subjects infected with the BA.1 and BA.2 Omicron VOCs differed significantly, perhaps because a smaller proportion of the population was booster-vaccinated during the BA.1 than during the BA.2 surge. 

Age, sex and rates of SARS-CoV-2 reinfection, hospitalization and death did not differ in patients infected with BA.2 and BA.1, in agreement with results reported for patients in Denmark [4]. In the absence of genomic sequencing, a 90-day timeframe is used to define a reinfection for surveillance purposes [25]. Therefore, all the reinfections described in the present study were caused by a VOC other than BA.1 or BA.2. A recent report from Norway suggested that infection with both BA.1 and BA.2 leads to an equally mild course of disease, with both being milder than after infection with the Delta variant [26]. The present study found, however, that patients infected with BA.2 were more likely to be symptomatic than those infected with BA.1, which may be associated with the slightly lower neutralizing antibody titers reported against BA.2 than against BA.1, with median titers against BA.2 being 1.3–1.4 fold lower than against BA.1 [24]. Moreover, the average time from the last vaccine dose to the day of infection was significantly longer in patients infected with BA.1 than BA.2, suggesting that the latter had escaped from vaccine protection. 

## 5. Conclusions

The identification of outbreaks caused by the SARS-CoV-2 BA.1 Omicron VOC demonstrated the high transmissibility and immune evasion of such a variant [9]. The rapid spread of the more transmissible BA.2 Omicron VOC, which may be the next prevailing SARS-CoV-2 VOC, suggests that, despite the end of the state of emergency in Italy, public health measures should not be discontinued, at least in the short term [11,27]. Further studies on new therapies against COVID-19 such as immunotherapies are also needed in order to expand the strategies to contain the spread of SARS-CoV-2 [28]. Non-pharmaceutical interventions, administration of booster doses and genomic surveillance should be maintained to mitigate the consequences of the spread of the Omicron VOCs.

## Figures and Tables

**Table 1:** Demographic and clinical characteristics of patients in the Apulia region of Italy infected with the BA.2 and BA.1 Omicron VOCs.

	BA.2		BA.1		
	*n*	%	*n*	%	*p*-Value
Total patients enrolled	284		175		
Sex					
Female	156	54.9%	94	53.7%	0.799
Male	128	45.1%	81	46.3%	
Age groups (years)					
0–4	13	4.6%	3	1.7%	0.122
5–11	19	6.7%	8	4.6%	0.877
12–17	14	4.9%	8	4.6%	0.862
≥18	238	83.8%	156	89.1%	0.111
Clinical status					
Asymptomatic	163	57.4%	138	78.9%	<0.00001
Mild	74	26.1%	31	17.7%	0.039
Moderate	35	12.3%	6	3.4%	0.0005
Severe	12	4.2%	0		
Chronic disease	9	3.2%	5	2.9%	0.85
Hospital admission	66	23.2%	29	16.6%	0.086
Deaths	5	1.7%	0		
Vaccination status					
Unvaccinated	72	25.4%	23	13.1%	0.024
Fully vaccinated with primary series	54	19.0%	103	58.9%	<0.00001
Fully vaccinated with primary series plus booster dose	158	55.6%	49	28.0%	<0.00001
Days from the last vaccine dose to infection (average, range)	99.1, 15–422		116.8, 17–317		0.01
Reinfections	15	5.3%	7	4.0%	0.532

## Data Availability

All data are available from the corresponding author by e-mail request.
